# Adsorption of ammonia on ZrO_*x*_-modified graphene nanoribbon: a first-principle investigation

**DOI:** 10.1007/s00894-022-05417-z

**Published:** 2022-12-22

**Authors:** Ahmad I. Ayesh, Maitha D. El-Muraikhi

**Affiliations:** Physics Program, Department of Mathematics, Statistics and Physics, College of Arts and Sciences, Qatar University, P. O. Box 2713, Doha, Qatar

**Keywords:** Graphene nanoribbon, ZrO_*x*_, Ammonia sensor, DFT

## Abstract

Ammonia (NH_3_) is a main environmental pollutant related to global warming, and reduction of its emission is the subject of multiple international agreements and regulations. Accordingly, the development of highly precise detectors to monitor its content in the environment is essential to track and limit its emission. This work examines the influence of modifying of armchair–graphene nanoribbon (AGNR) by zirconium (Zr) and its oxides on its adsorption for NH_3_ gas. Density functional theory (DFT) computations are utilized to investigate the band structure, adsorption energy (${E}_{d}$), adsorption length ($D$), charge transferred ($\Delta Q$), and density of states (DOS) of pristine and modified structures with ZrO_*x*_ ($x=0, 1, \mathrm{or }2$). ZrO_*x*_ is presented to AGNR nanostructure by two pathways: substitution of carbon atoms (doping) and introduction on top of the AGNR surface (decoration). The findings of the investigation illustrate great improvement of NH_3_ adsorption on AGNR due to its modification. Although the adsorption energy is enhanced in general upon modification, AGNR structures where ZrO_*x*_ substitute carbon atoms exhibit greater adsorption energy as compared with the decoration scheme. The maximum energy of adsorption is for the AGNR structure doped with ZrO_2_, followed by that doped with Zr. The adsorption energy of NH_3_ on the ZrO_2_-doped AGNR is − 10.05 eV with an adsorption length of 2.4 Å and − 0.214e charge transferred. As compared to the pristine structure, the adsorption energy for NH_3_ on AGNR doped with ZrO_2_ increases 22.2 times. Therefore, AGNR nanostructure doped with ZrO_*x*_ can be considered for practical sensors for the applications of detection and control of ammonia emission.

## Introduction

Agricultural activities result 80–90% of the total global ammonia (NH_3_) emission [1]. Its emission results mainly through volatilization of livestock manure as well as a synthetic mineral for nitrogen based fertilizers [2, 3]. NH_3_ emissions alongside with other greenhouse gases that result mainly from agricultural activities have negative effects on air quality [4]. It is associated with two main environmental hazards: acidification and eutrophication [5]. When ammonia interacts with water, it increases the acidification of both soil and water [6]. Furthermore, the deposition of ammonia in soil and water can increase their nitrogen level that may increase the eutrophication of aquatic ecosystems [5]. Human exposure to ammonia concentrations of 25 ppm or higher may negatively influence the lung functions and respiratory track, and it may result injuries and risking burns for high exposure concentrations [7]. Accordingly, precise environmental monitoring of ammonia level and control of its emission (especially at the industrial production plants such as production plants of plastics, fabrics, and explosives) are essential [8].

Conductometric chemical sensors are electronic devices that detect the presence of selected chemicals and their concentrations at their surroundings through the change in the electrical conductance [9–13]. The chemical sensors are important for domestic safety in addition to environmental assessment because they identify hazardous gas concentrations, and send their values to safety as well as control systems.

Graphene is known as a two dimensional material of a mono layer of carbon atoms in a lattice structure of honeycomb [14, 15]. Graphene exhibits two main structures following the arrangement of its atoms: zigzag (ZGNR) and armchair (AGNR). The two structures have different energy band structures where ZGNR is categorized as a conductor whereas AGNR can be either conductor or semiconductor [16–18]. Graphene is considered a material with extraordinary characteristics that include excellent thermal conduction, optical transparency, alongside its high density that inhibits its penetration by gases [19]. Experimentally, the band gap of AGNR can be controlled through decreasing its dimensions to one or quasi one-dimensional [20]. AGNR may be classified into three categories depending on the quantity of dimers through a nanoribbon line (${N}_{d}$), with ${N}_{d}=3n, 3n+1, or 3n+2$, with $n$ is an integer. The first two categories are semiconductor, whereas the last one is metallic [21]. The semiconductor AGNR is more attractive for device applications such as optical devices, biodevices, and chemical sensors [12, 22, 23]. Additionally, it is investigated heavily for gas sensor applications due to its ability to adsorb various gases [18, 24–26]. The sensitivity as well as the selectivity may be promoted by modification of AGNR surface, i.e., by (i) doping that involves replacement of selected carbon atom(s) by dopant atom(s) and (ii) decoration that involves precise addition of atom(s) on its surface [23, 27, 28].

The effect of modifying graphene nanoribbon has been investigated intensively recently for gas sensing utilization [29], since its impact on enhancing AGNR adsorption features was illustrated experimentally [12, 19]. Miao et al. investigated the decomposition of ammonia on graphene modified by metal clusters of Ni_6_, Co_6_, and Fe_6_ using DFT computations [30]. They found that graphene modified with Ni_6_ is more active for ammonia adsorption and dissociation. Barkov examined modifying graphene nanoribbons with carboxylic acid (COOH) group and its effect on ammonia adsorption using DFT calculations [31]. They found that wet nanoribbons interact further with ammonia. The carboxyl group attracts water molecule that is favorable in terms of energy that in turn promotes ammonia adsorption where COOH and water represent adsorption centers for NH_3_. VE C. Padilla et al. examined ammonia adsorption on graphene doped with P and Si atoms by means of DFT computations [32]. Phosphorus-modified graphene exhibits metallic behavior, while the silicon-modified graphene is a semiconductor (band gap ~ 0.25 eV). The phosphorus-modified structure was found to be more favorable for ammonia adsorption than the silicon-modified one.

Herein, the effect of modification of AGNR (${N}_{a}=3p$) by ZrO_*x*_ ($x=0, 1, \mathrm{or }2$) on its adsorption for ammonia gas is investigated using first principles and DFT computations [33]. The objective of this work is to explore the influence of AGNR doping and surface decoration by ZrO_*x*_; thus, AGNR structures are modified by ZrO_*x*_ species through either substitution of carbon atom(s) by ZrO_*x*_ (doping), or addition of ZrO_*x*_ on the central position of AGNR surface (decoration). The adsorption of ammonia on pristine and modified graphene structures is explored by detailed investigation of band structure, adsorption energy (${E}_{\mathrm{d}}$), adsorption length ($D$), charge transferred ($\Delta Q$), and density of states (DOS).

## Simulation methods

Kohn–Sham (KS) DFT calculations were employed to explore the influence of ZrO_*x*_ modification of AGNR structures on their adsorption of NH_3_ [34]. The different structures were labeled as presented in Table 1. The computations utilized a framework that consists of a linear combination of atomic orbitals (LCAO) along with pseudopotential perturbation. The electrons are modeled as a system of non-interacting gas that exhibits a density ($n$) within an effective potential (${V}^{\mathrm{eff}}\left(n\right)$) according to the equation [35]:

**Table 1:** AGNR structures and their descriptions alongside with the band gap energy (*E*_g_) prior to NH_3_ adsorption

Structure	Description	*E*_g_ (eV)
AGNR	Pristine AGNR structure	0.7122
Zr on AGNR	AGNR structure decorated with Zr	0.2516
ZrO on AGNR	AGNR structure decorated with ZrO	0.4404
ZrO_2_ on AGNR	AGNR structure decorated with ZrO_2_	0.2471
Zr + AGNR	AGNR structure doped with Zr	0.4596
ZrO + AGNR	AGNR structure doped with ZrO	0.0775
ZrO_2_ + AGNR	AGNR structure doped with ZrO_2_	0.0579

1 $${V}^{\mathrm{eff}}\left(n\right)={V}^{H}\left(n\right)+{V}^{xc}\left(n\right)+{V}^{\mathrm{ext}}\left(n\right)$$

The electrostatic interaction amongst electrons is represented by the Hartree potential (${V}^{H}$). The exchange–correlation (XC) potential is represented by (${V}^{xc}$), whereas the electrostatic potential energy of electrons is indicated by (${V}^{\mathrm{ext}}$).

The Hamiltonian of KS (${\widehat{H}}^{\mathrm{KS}}$) may be presented as [35]

2 $${\widehat{H}}^{\mathrm{KS}}=-\frac{{\hslash }^{2}}{2m}{\nabla }^{2}+{V}^{\mathrm{eff}}$$

where $m$ is the mass of electron and $\mathrm{\hslash }=\frac{h}{2\pi }$ ($h$ is Planck’s constant). The KS equation is represented by a DFT-LCAO process through numerical values to permit effective implementation of KS-DFT computations for all different structures. Two types of approximations were implemented within the KS-DFT formalism: local density (LDA) alongside with generalized gradient (GGA). Their implementation was according to Perdew–Burke–Ernzerhof (PBE) scheme combined with Grimme approximations (DFT-D2) that account for Van der Waals force [36, 37]. The above estimates were selected because of their reasonable consideration of both accuracy and efficiency of DFT computations as presented by various reports [38].

A simulation package from Synopsys: Quantum ATK associated with a virtual nanolab (VNL) was utilized to perform the DFT calculations. Single sheets of AGNR with the edge atoms terminated by hydrogen were used in this investigation [39]. Zr, ZrO, and ZrO_2_ (referred as ZrO_*x*_) were used to modify the structures either by doping or by decoration. Prior to any computations, an LBFGS optimizer was employed to optimize the structures [40], and they were relaxed where the residual force per atom and stress tolerance were under 0.05 eV/Å and 0.1 GPa, correspondingly. The $k$-point sampling mesh was 4 × 2 × 1, with a mesh density of 100 Hartree. This $k$-point sampling mesh was selected since it provided accurate results with reasonable computation time. The binding energy of ammonia can be evaluated by calculation of adsorption energy for a structure was evaluated using [17, 18, 41]:

3 $${E}_{d}={E}_{\mathrm{structure}+{\mathrm{NH}}_{3}}-({E}_{\mathrm{structure}}+{E}_{{\mathrm{NH}}_{3}})$$

Herein, ${E}_{\mathrm{structure}+{\mathrm{NH}}_{3}}$ is the total energy of AGNR structure with adsorbed NH_3_, ${E}_{\mathrm{structure}}$ is the total energy of an unexposed AGNR structure, and ${E}_{{\mathrm{NH}}_{3}}$ is the total energy for NH_3_ molecule. The value of ${E}_{d}$ is an indication of the suitability of a structure to adsorb NH_3_: the further the negative ${E}_{d}$, the more appropriate the structure for NH_3_ adsorption. The charge relocation among NH_3_ and a structure is another indicator of preference for its adsorption, and it was evaluated by the Mulliken method as [20, 23]

4 $$\Delta Q={q}_{f}-{q}_{0}$$

where ${q}_{f}$ and ${q}_{0}$ denote the final and initial Mulliken charges of the gas, respectively. If electros were moved from NH_3_ to AGNR, $\Delta Q$ will be negative.

## Results and discussion

**Fig. 1:**
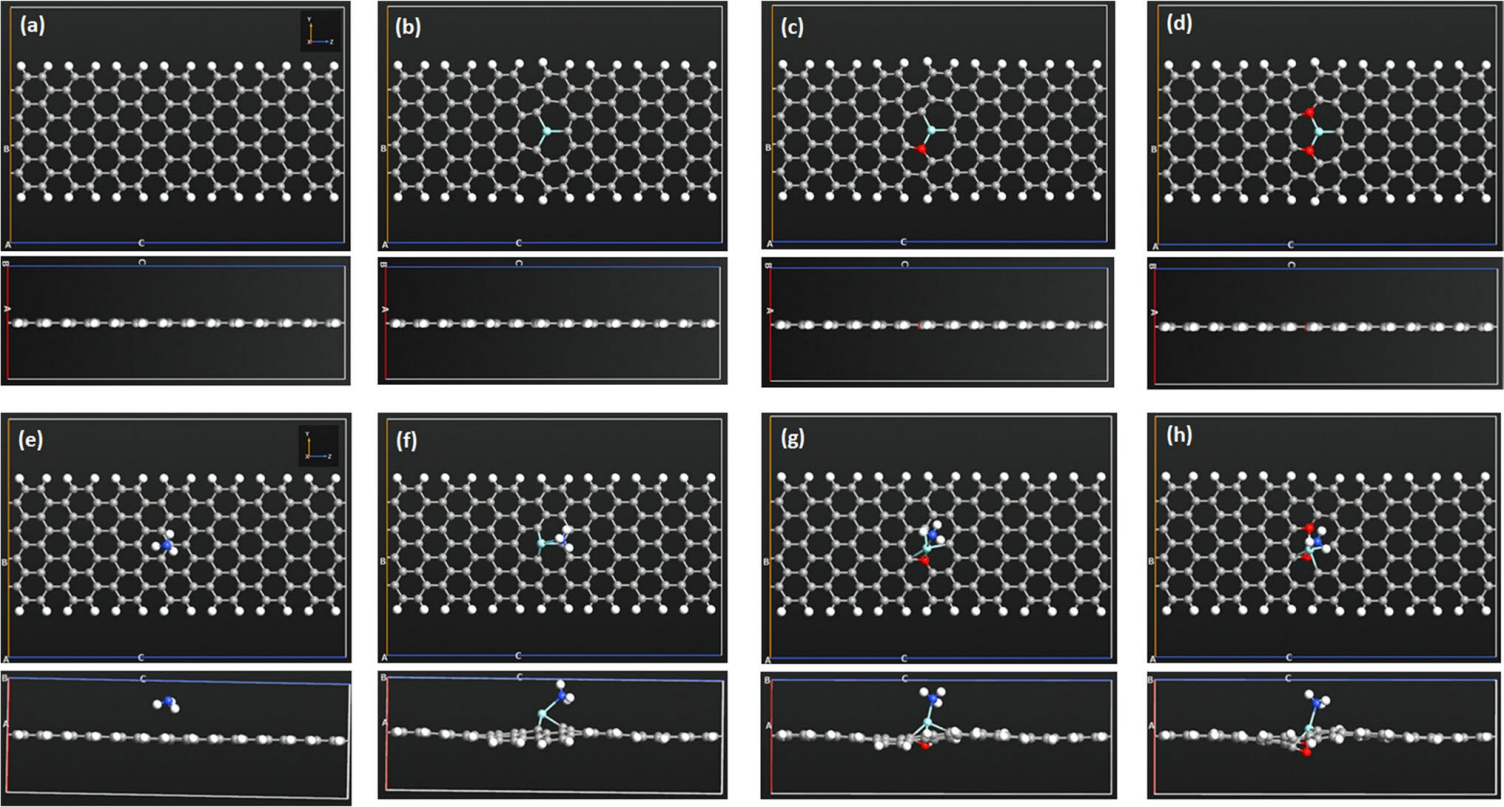
Optimized AGNR structures (top and side views) prior (**a**–**d**) and post (**e**–**h**) to NH_3_ adsorption: (**a**, **e**) AGNR, (**b**, **f**) doped Zr + AGNR, (**c**, **g**) doped ZrO + AGNR, and (**d**, **h**) doped ZrO_2_ + AGNR

**Fig. 2:**
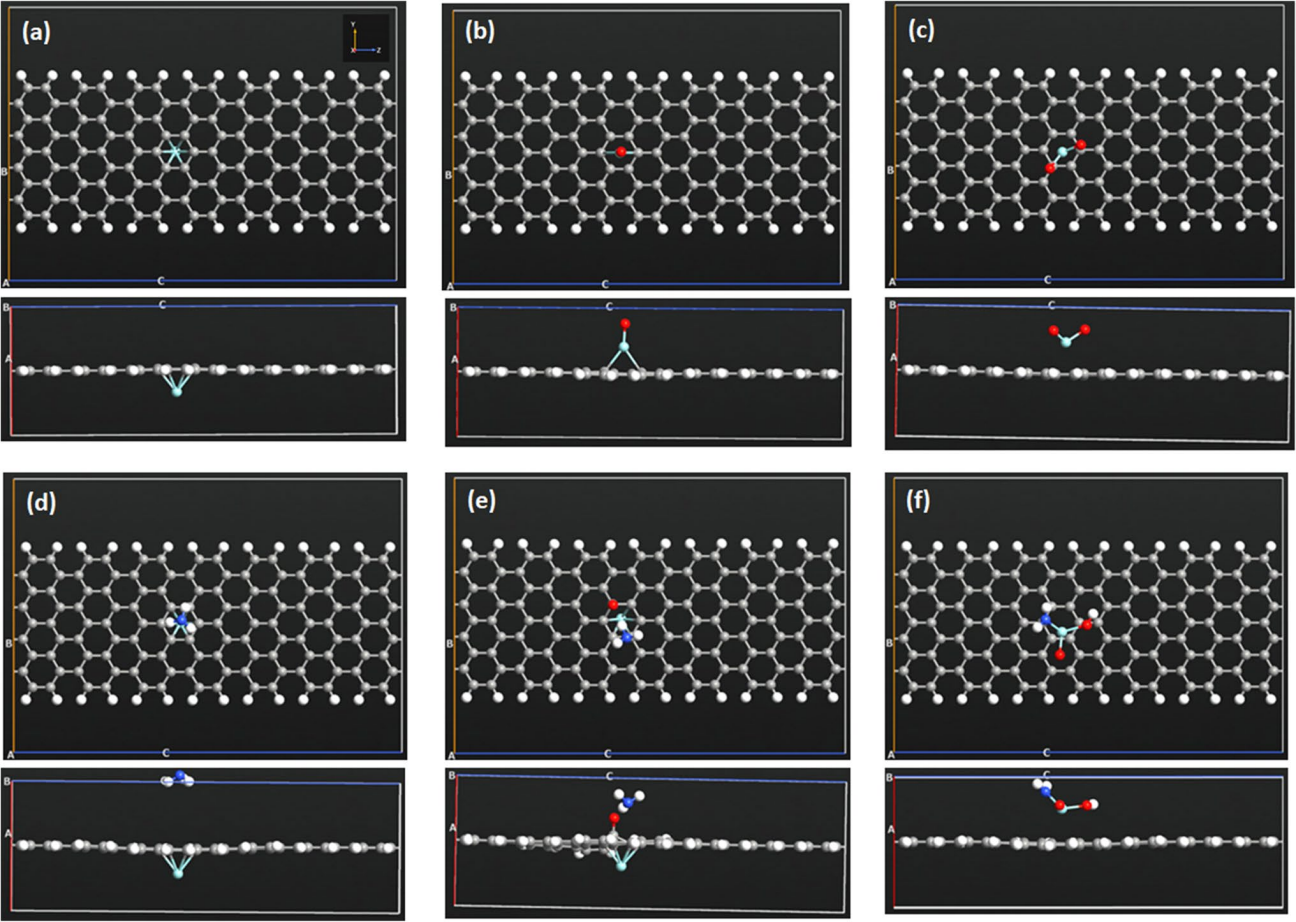
Optimized AGNR structures (top and side views) prior (**a**–**c**) and post (**d**–**f**) to NH_3_ adsorption: (**a**, **d**) decorated Zr on AGNR, (**b**, **e**) decorated ZrO on AGNR, and (**c**, **f**) decorated ZrO_2_ on AGNR

The effect on the adsorption capacity for ammonia gas of AGNR structures modified by ZrO_*x*_ is examined. The modification is established either by doping or decoration for AGNR structure. The detailed description of the different examined nanostructures is presented in Table 1. The table also reveals the band gap energy (${E}_{g}$) of the different structures. The maximum value of ${E}_{g}$ is for the pristine structure, while the modified structures exhibit lower values of ${E}_{g}$. AGNR structures doped with ZrO and ZrO_2_ demonstrate the lowest ${E}_{g}$ values. The optimized doped AGNR structures are presented in Fig. 1a–d. The carbon atoms are rearranged as a result of energy optimization near the doping site. The edge atoms of AGNR structures are passivated using hydrogen atoms to avoid reconstruction of the structures as a result of satisfying dangling bonds for the edge atoms [39]. The modification cites of ZrO_*x*_ are selected at the center of the structures to minimize edge effect, where many cites reveal similar results because of the high symmetry of nanoribbons. The C–C bond length of pristine AGNR is 1.42–1.43 Å. The doped structures exhibit the following bond lengths: (i) Zr + AGNR structure: the Zr-C bond length is 1.89–1.90 Å; (ii) ZrO + AGNR structure: the Zr-C bond length is 1.86–1.87 Å, the Zr-O bond length is 1.90 Å, and the O-C bond length is 1.42–1.44 Å; (iii) ZrO_2_ + AGNR structure: the Zr-C bond length is 1.83 Å, the Zr-O bond length is 1.89 Å, and the O-C bond length is 1.42–1.44 Å. The bond lengths are as follows for the decorated structures: (i) Zr on AGNR structure, the Zr-C bond length is 2.30 Å; (ii) ZrO on AGNR structure, the Zr-C and Zr-O bond lengths are 2.41–2.44 Å and 1.78 Å, respectively; and (iii) ZrO_2_ on AGNR structure, the Zr-C and Zr-O bond lengths are 2.75–2.95 Å and 1.82 Å, correspondingly. Doping an AGNR structure with ZrO_*x*_ by substitution of carbon atoms produces a stress on the structure that results modifications of the bond lengths for C-Zr, Zr-O, and C-O to relief the stress [42]. The optimized decorated structures are presented in Fig. 2a–c. Both Zr and ZrO are bonded to interstitial locations with the structures that minimize their overall energy. Nevertheless, ZrO_2_ remains on top of the structure unbounded due to its high stability as compared with both Zr and ZrO [43].

**Fig. 3:**
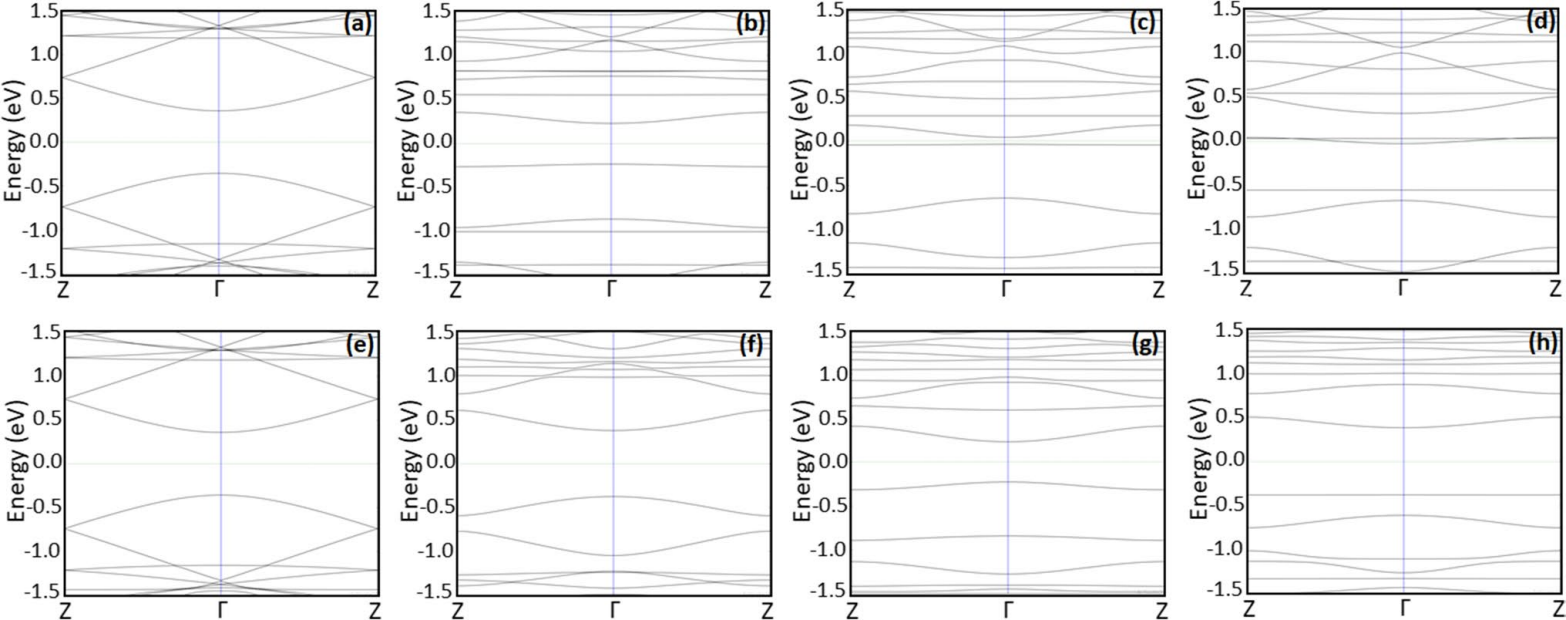
Band structures for the optimized AGNR structures prior (**a**–**d**) and post (**e**–**h**) to NH_3_ adsorption: (**a**, **e**) AGNR, (**b**, **f**) doped Zr + AGNR, (**c**, **g**) doped ZrO + AGNR, and (**d**, **h**) doped ZrO_2_ + AGNR

**Fig. 4:**
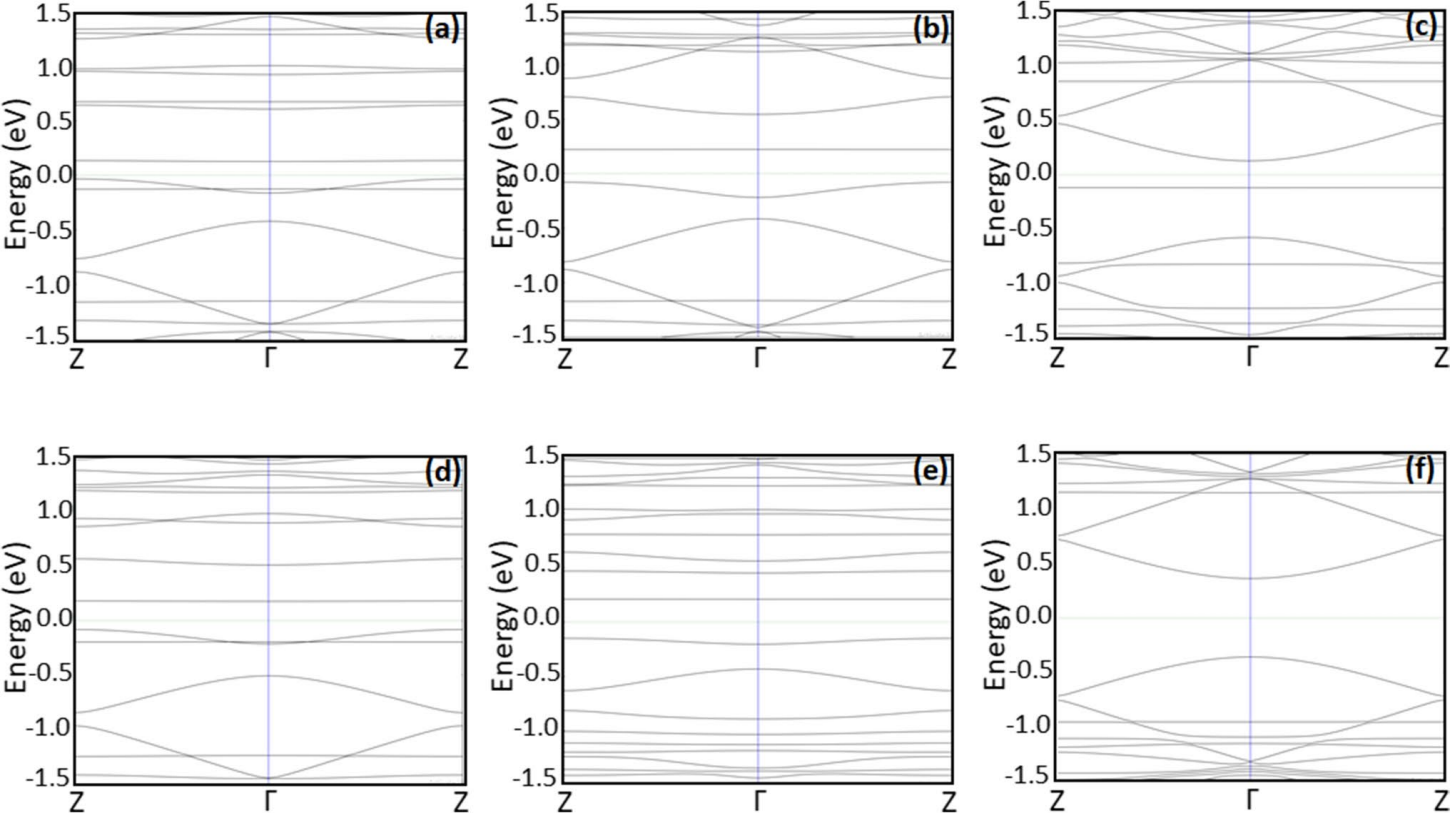
Band structures for the optimized AGNR structures prior (**a**–**c**) and post (**d**–**f**) to NH_3_ adsorption: (**a**, **d**) decorated Zr on AGNR, (**b**, **e**) decorated ZrO on AGNR, and (**c**, **f**) decorated ZrO_2_ on AGNR

The effect of AGNR structure modification on the band structure is presented in Fig. 3b–d for doped structures and Fig. 4b–d for decorated structures. The band structure of the pristine AGNR is presented in Figs. 3a and 4a. The figures demonstrate that al the band structures have a parabolic form near the Γ point. The evaluated density of states within the band structures within both valance and conduction bands increases as a result of modification (for both modified structures, doped and decorated) as compared with the unmodified structure. The modification leads to the introduction of new bands near the Fermi level, and shift of the valance band close to the Fermi level in agreement with the results presented in Table 1.

### Adsorption energy

Figure 1e–h reveals the doped AGNR structures post to adsorption of NH_3_ gas. The figures illustrate that NH_3_ molecule is unbonded to the pristine structure; however, it is bonded to all other ZrO_*x*_-doped AGNR structures. Upon introduction of an NH_3_ gas molecule to an AGNR structures, where the molecule orientation is set during the optimization stage to exhibit the minimum energy leading to the maximum stable configuration, NH_3_ gas is adsorbed on AGNR through physisorption and chemisorption. Physisorption involves weak attraction force among the gas and AGNR, while chemisorption involves strong chemical bonding among the gas and AGNR [44, 45]. In case of Pristine-Gr substrate, NO and NO_2_ molecules are physisorbed on its surface while in case of BC2P-Gr and BCP2-Gr, both NO and NO_2_ molecules are chemisorbed on substrate.

The chemisorption of NH_3_ gas on the ZrO_*x*_-doped AGNR structures indicates that they are favorable for NH_3_ adsorption unlike the pristine structure. The decorated AGNR structures post to adsorption of NH_3_ gas are presented in Fig. 2d–f. The figures reveal that NH_3_ molecule is not chemisorbed on any decorated AGNR structure, i.e. no bond can be observed between NH_3_ gas and the structures.

### Band structure

The effect of ammonia adsorption on AGNR structures on the band structure is demonstrated in Figs. 3a and 4a for pristine structure, Fig. 3e–h for doped structures, and Fig. 4e–h for decorated structures. Further, bands appear as a result of NH_3_ gas adsorption and, in general, the band gap increases after adsorption as presented in Table 2. The modifications in the band structure post to NH_3_ adsorption and the introduced bands are indicators of the generation of extra electronic states as a result of adsorption [32]. The developments appear within the band structure specify that the ZrO_*x*_ modification of AGNR is an effective approach for adsorption of NH_3_ molecule.

**Table 2:** Band gap energy, adsorption energy, ammonia adsorption length, and charge transferred among the gas and the structures post to adsorption of NH_3_

Structure	*E*_g_ (eV)	*E*_d_ (eV)	*D* (Å)	∆*Q* (e)
NH_3_ + AGNR	0.7156	− 0.4518	3.4300	− 0.0060
NH_3_ + Zr on AGNR	0.3743	− 1.9832	5.2900	− 0.2400
NH_3_ + ZrO on AGNR	0.4143	− 4.2570	3.0500	− 0.0310
NH_3_ + ZrO_2_ on AGNR	0.7212	− 2.4664	3.3100	0.0970
NH_3_ + Zr + AGNR	0.7536	− 9.1002	2.4200	− 0.2350
NH_3_ + ZrO + AGNR	0.4575	− 8.8799	2.4000	− 0.2250
NH_3_ + ZrO_2_ + AGNR	0.7625	− 10.0513	2.4200	− 0.2140

### Density of states

**Fig. 5:**
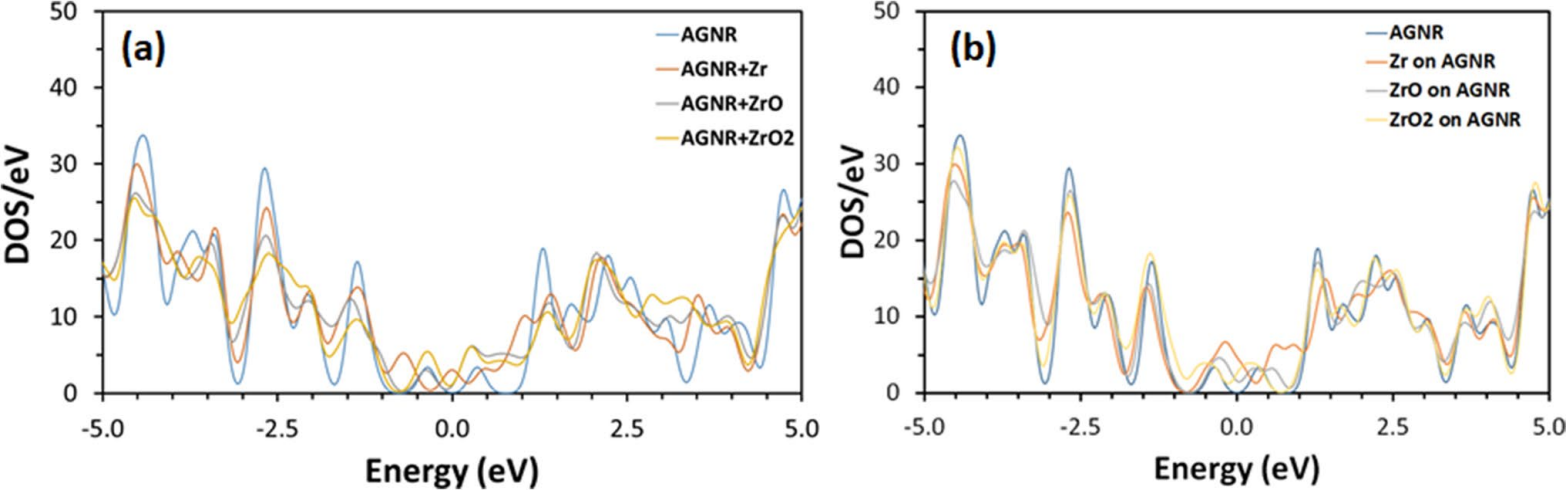
Electronic density of states of both (**a**) doped and (**b**) decorated AGNR structures before adsorption of NH_3_ gas

**Fig. 6:**
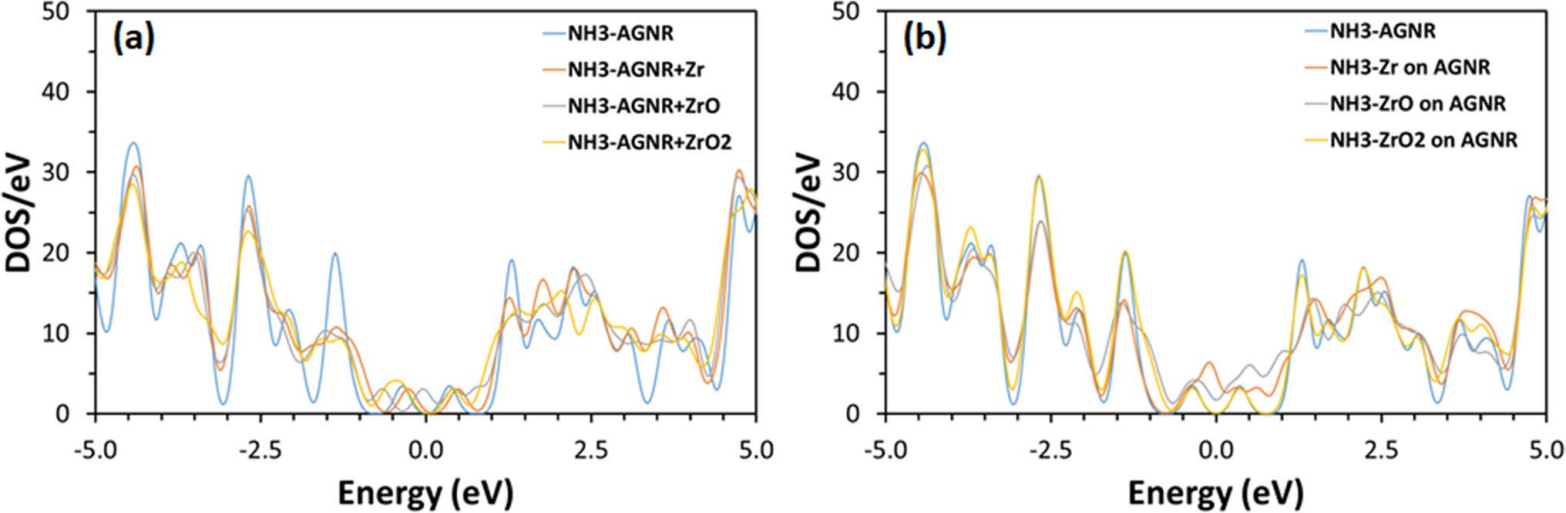
Electronic density of states of both (**a**) doped and (**b**) decorated AGNR structures after adsorption of NH_3_ gas

The effect of AGNR doping on band structure is examined using DOS as presented in Fig. 5a. The figure illustrates that DOS decreases generally due to doping, and new bands appear within both conduction and valance bands. For example, the following bands appear as a result of doping at: 1.06, 3.52, − 0.65, and − 4.00 eV for the Zr-doped AGNR; and 0.726, 3.48, and − 1.43 eV for the ZrO-doped AGNR; and 0.726, 2.85, 3.33, − 3.63, and − 4.26 eV for the ZrO_2_ -doped AGNR. Decoration of AGNR modifies the DOS as illustrated in Fig. 5b. Although the intensity of DOS is lower for the decorated structures as compared with the pristine structure, it is higher than the equivalent for the case of doping. New bands appear within both conduction and valance bands of decorated AGNR such as at 0.67, 0.93, 1.37, 1.85, and − 0.19 eV for the Zr-doped AGNR; 0.36, 0.61, 1.96, and − 0.24 eV for the ZrO-doped AGNR; and 0.22, 1.69, and − 1.52 eV for the ZrO_2_-doped AGNR. Figure 5 reveals that the DOS of both doped and decorated structures increases near the Fermi level, in agreement with the results of Figs. 3 and 4. In addition, the conduction band exhibits lower DOS than the valence band for all AGNR structures. Figure 6 shows the effect of NH_3_ adsorption on DOS for the different ZrO_*x*_-modified AGNR sheets. The DOS preserves its overall features prior to adsorption of NH_3_ gas. The DOS near the Fermi level is higher for the decorated structures as compared with the doped structures. Adsorption of NH_3_ decreases the intensity of DOS of many bands for the doped AGNR structures, such as at 1.28, − 1.34, − 2.69, and − 4.42 eV. Nevertheless, lower decrease in the DOS is observed for the decorated AGNR structures. The results of DOS designate that many electronic states are available within the AGNR structures post to NH_3_ adsorption [46]. Accordingly, the ZrO_*x*_-modified AGNR structures can be considered as effective systems for NH_3_ gas adsorption.

## Discussion

The adsorption capacity for NH_3_ of AGNR modified by ZrO_*x*_ is explored using adsorption energy, charge transferred to the NH_3_ molecule, along with the adsorption length as shown in Table 2. The table demonstrates that the modified AGNR structures have greater adsorption energies in comparison with the unmodified AGNR, indicating that ZrO_*x*_ modification of AGNR structures enhances their NH_3_ adsorption. The adsorption energy of the doped structures is higher than its equivalent of the decorated structures. In fact, all doped structure are suitable for NH_3_ adsorption. Herein, ammonia molecule bonds to the ZrO_*x*_ dopant which is already bonded to AGNR structure. Nevertheless, the highest adsorption energy for NH_3_ is for the ZrO_2_ + AGNR-doped structure, where it is 22.2 times greater than that of the unmodified AGNR structure. Furthermore, the adsorption length results are in agreement with those of the adsorption energy. Here, all doped structures exhibit low adsorption length of ~ 2.4 Å indicating strong bonds among NH_3_ and the structures. The adsorption length is higher for the decorated structures, in comparison with the doped structures, which is in agreement with the observation (Fig. 2), i.e., no chemisorption is established for those structures. The charge transferred from/to ammonia molecule for the doped structures is ~ -0.2e which is almost similar for the three structures, and it is higher than that of decorated structures (except that for the Zr decoration case). The negative sign indicates that the charge is transferred from NH_3_ to AGNR structures. The considerable amount of transferred charge from NH_3_ molecule to the doped structures specifies the formation of chemical bonds between them. The charge transfer observation indicates that adsorption of NH_3_ molecule on doped AGNR structures modify their electronic features notably (in agreement with DOS in Fig. 6) to generate strong interactions among NH_3_ and the AGNR structures resulting high energy of adsorption [47]. The charge transferred among NH_3_ and the ZrO_*x*_-modified AGNR structures are extracted within AGNR [12]. On the other hand, the low magnitude of charge transferred among NH_3_ and the decorated AGNR structures is allocated to the fact that NH_3_ is not chemisorbed on either of them.

The adsorption energy towards ammonia of the doped AGNR structure is boosted due to ZrO_2_ modification, for instance, it is ~ 41 times greater than that presented for zigzag graphene nanoribbon modified with OH [20]. The illustrated results indicate that the ZrO_*x*_-doped structures are more favorable for NH_3_ adsorption than the decorated ones [48–50]. Accordingly, one can conclude that although the small differences in the adsorption energy for the doped structures, their values are close, signifying that doping AGNR with Zr metal and its oxides is a suitable approach for adsorption of NH_3_. Additionally, if decoration is considered for AGNR adsorption of NH_3_, the ZrO decorated structure is the suitable one due to its relatively high adsorption energy. The improved NH_3_ adsorption on doped AGNR structures can be allocated to the high affinity of ZrO_x_ to the gas upon doping [51]. Here, the reaction of both Zr^1+^ and Zr.^2+^ with nitrogen and oxygen atoms is favorable [52]. Doping AGNR structures with ZrO_*x*_ improves their reactivity by supplying additional negative charges for the delocalized π bond due to the low electronegativity of ZrO_x_ in comparison with carbon atoms [42]. The low ammonia adsorption energy of decorated AGNR structures in comparison with the doped ones may be allocated to the weak hybridization among their overlapped orbitals: 3p for nitrogen, and 3d and 4 s for zirconium [53]. On the other hand, the 3p orbital of nitrogen, 3d orbital of zirconium, and 2p orbital of oxygen exhibit strong hybridization for the doping case [53]. The influence of zirconium and its oxides on enhancing ammonia adsorption is examined and presented experimentally [54]. This is assigned to trapping of electrons within the conduction band that generates electron depletion region. NH_3_ molecule reacts with the adsorbed ions of oxygen, causing the release of the trapped electrons and narrowing the space charge [54]. The adsorption of NH_3_ is enhanced by the transfer of charge due to the presence of oxygen ions on the AGNR surface which may be presented as [55]

$${4\mathrm{NH}}_{3}+3{\mathrm{O}}_{2}^{-}\left(\mathrm{ads}\right)\to {2\mathrm{N}}_{2}+6{\mathrm{H}}_{2}\mathrm{O}+6{\mathrm{e}}^{-}$$

The equation reveals that NH_3_ adsorption generates additional charges as demonstrated by the charge transferred (Table 2). In all doped structures, NH_3_ is bonded to the Zr atom. The reaction of NH_3_ with zirconium can be presented as below [56]:

$${\mathrm{NH}}_{3}+\mathrm{Zr}\to {\mathrm{ZrNH}}_{3}$$

However, for decorated structures, the bond of Zr atom are established with carbon rather than the ammonia. The present value of adsorption energy for ZrO_2_ + ZGNR structure towards NH_3_ is compared with latest results for NH_3_ adsorption of graphene-based structures, as presented in Table 3. The table demonstrates the major improvement of the present work for ammonia adsorption due to ZrO_2_ doping.

**Table 3:** Recent reported values for the adsorption energy of NH_3_ on graphene-based structures

Structure	*E*_d_ (eV)	Reference
ZrO_2_ + AGNR	− 10.0513	This work
Co-AGNR	− 1.46	[57]
Pt-AGNR	− 1.816	[58]
N-AGNR	− 0.304	[59]
OH-modified zigzag graphene nanoribbon	− 0.244	[20]
Ce-modified graphene	− 0.8	[60]

## Conclusion

First-principle computations of density functional theory (DFT) were employed to explore the effect of modification of nanoribbon with armchair-graphene structure (AGNR) by ZrO_*x*_ ($x=0, 1,\mathrm{ or} 2$) on its adsorption of ammonia (NH_3_) gas. The modification of AGNR structures was established either by doping (i.e., substitution of carbon atom(s)), and decoration on the surface. Ammonia adsorption was examined by thorough investigation of the adsorption energy (${E}_{d}$), band structure, charge transferred ($\Delta Q$), adsorption length ($D$), and density of states (DOS). The findings reveal that NH_3_ adsorption was greatly enhanced upon modification with ZrO_*x*_; nevertheless, decoration was less effective than doping. The highest adsorption energy of NH_3_ was found for the structure doped by ZrO_2_, followed by that doped by Zr. Adsorption of NH_3_ on the ZrO_2_ doped AGNR exhibits a favorable adsorption of ${E}_{d}\sim -10.05 eV$, $D\sim 2.4 \dot{\mathrm{A}}$, and $\Delta Q\sim -0.214e$. The energy of adsorption for the AGNR structure doped with ZrO_2_ for NH_3_ gas was 22.2 times greater than that of the undoped structure. Moreover, AGNR structures doped with Zr and ZrO have also exhibited high adsorption energy for NH_3_. The results of this investigation demonstrate that doping of AGNR with ZrO_*x*_ is a high potential approach towards production of sensitive NH_3_ sensors.

## Data Availability

The raw data required to reproduce these findings are available on request from the author.
